# The impact of COVID-19 pandemic on aortic valve surgical service: a single centre experience

**DOI:** 10.1186/s12872-021-02253-6

**Published:** 2021-09-14

**Authors:** Dimitrios Vlastos, Ishaansinh Chauhan, Kwabena Mensah, Maria Cannoletta, Athanasios Asonitis, Ahmed Elfadil, Mario Petrou, Anthony De Souza, Cesare Quarto, Sunil K. Bhudia, Ulrich Rosendahl, John Pepper, George Asimakopoulos

**Affiliations:** Royal Brompton and Harefield NHS Foundation Trust, London, UK

**Keywords:** COVID-19, Aortic valve surgery, Service evaluation, Adult cardiac surgery, Pandemic

## Abstract

**Background:**

The coronavirus-disease 2019 (COVID-19) pandemic imposed an unprecedented burden on the provision of cardiac surgical services. The reallocation of workforce and resources necessitated the postponement of elective operations in this cohort of high-risk patients. We investigated the impact of this outbreak on the aortic valve surgery activity at a single two-site centre in the United Kingdom.

**Methods:**

Data were extracted from the local surgical database, including the demographics, clinical characteristics, and outcomes of patients operated on from March 2020 to May 2020 with only one of the two sites resuming operative activity and compared with the respective 2019 period. A similar comparison was conducted with the period between June 2020 and August 2020, when operative activity was restored at both institutional sites. The experience of centres world-wide was invoked to assess the efficiency of our services.

**Results:**

There was an initial 38.2% reduction in the total number of operations with a 70% reduction in elective cases, compared with a 159% increase in urgent and emergency operations. The attendant surgical risk was significantly higher [median Euroscore II was 2.7 [1.9–5.2] in 2020 versus 2.1 [0.9–3.7] in 2019 (*p* = 0.005)] but neither 30-day survival nor freedom from major post-operative complications (re-sternotomy for bleeding/tamponade, transient ischemic attack/stroke, renal replacement therapy) was compromised (*p* > 0.05 for all comparisons). Recommencement of activity at both institutional sites conferred a surgical volume within 17% of the pre-COVID-19 era.

**Conclusions:**

Our institution managed to offer a considerable volume of aortic valve surgical activity over the first COVID-19 outbreak to a cohort of higher-risk patients, without compromising post-operative outcomes. A backlog of elective cases is expected to develop, the accommodation of which after surgical activity normalisation will be crucial to monitor.

## Introduction

The severe acute respiratory syndrome coronavirus 2 and the attendant coronavirus-disease 2019 (COVID-19) emerged in December 2019, resulting in a pandemic declaration by the World Health Organisation by March 2020 [1]. By the end of May 2020 more than 6 million cases and 374,000 fatalities had been reported worldwide; for the United Kingdom, the reported incidence was 90,000 and almost 10,000 respectively [2]. This has imposed an unprecedented burden on the provision of healthcare services in general, and surgical treatment specifically [3]. The postponement of elective cases and the redistribution of workforce and resources reshaped the dynamics of surgical activity [3, 4].

Our aortic surgery team, among other cardiac surgical teams, had the task to achieve a delicate balance between patients whose treatment could be safely postponed, versus patients with life-threatening advanced chronic or emergency disease, in the context of severely limited intensive care resources availability [5, 6]. On the one hand, Pan-London Emergency Cardiac Surgery (PLECS) protocol facilitated this by providing a centralised pathway to COVID-19 protected surgical facilities [7]. On the other hand, the correlation of cardiovascular risk factors with worse COVID-19 outcomes [8–10] as well as the occasionally unpredictable trajectory of aortic valve disease [11] further complicated this process. Moreover, surgical theatre availability in our institution was severely compromised during the first three months of our pandemic response, since one of the two sites served as an exclusive COVID-19 Extracorporeal Membrane Oxygenation (ECMO) referral centre. Operative activity was restored at both sites thereafter, significantly enhancing our surgical volume capability.

### Aims and objectives

The aim of this service evaluation report is to provide an objective assessment of the effects of COVID-19 pandemic on the cumulative aortic valve surgical activity at our institution. More specifically, the number of operations undertaken between March and May 2020 -via the modified cardiac surgery pathway- were compared with the respective activity during the period from March 2019 to May 2019. A similar comparison was performed with our activity between June 2020 and August 2020, when our surgical volume capability was enhanced by re-commencement of operations at both sites included in our institution. In addition, a more detailed analysis regarding the differential impact on elective versus urgent or emergency cases, as well as on patients with mild clinical disease versus severely symptomatic ones was conducted. We also investigated how surgical mortality and major post-operative complication rates were affected, especially given the self-explaining prioritization of severe and urgent/emergency cases. Lastly, we assessed the effectiveness of our COVID-19 screening protocol as denoted by the comparison of the pre- and post-operative COVID-19 status of our patients.

## Methods

### Study design and population

This was a retrospective study (service evaluation project) conducted at the Royal Brompton and Harefield NHS Foundation trust. It included a total of 384 adult patients (mean age = 66.5 ± 13.5 years, 68.2% male) undergoing aortic valve surgery, either isolated or with concomitant procedures, for a primary aortic valvular disease indication during the three studied periods (namely March–May 2019, March–May 2020, and June–August 2020). Patients undergoing aortic valve replacement (AVR) for incidental valvular disease diagnosed at pre-operative workup were excluded.

### Statistical analysis

Data were extracted from the local surgical database and analysed using the SPSS v20 software. They included the demographics and clinical characteristics of patients treated over the periods of interest, type of operations and their indication, pre- and post-operative COVID-19 status, as well as major post-operative complications, namely re-sternotomy for bleeding or tamponade, transient ischemic attack (TIA) or stroke, new need for renal replacement therapy (RRT), and 30-day mortality. Data with a non-gaussian distribution were expressed as median (interquartile range) and were analysed after transformation into ranks. Chi-square Fisher exact test was used to compare categorical clinical characteristics and outcomes during the two investigated periods. Independent sample *t*-test was utilised for parametric ordinal data. In all analyses, we used two tailed tests with *p* < 0.05.

## Results

**Fig. 1:**
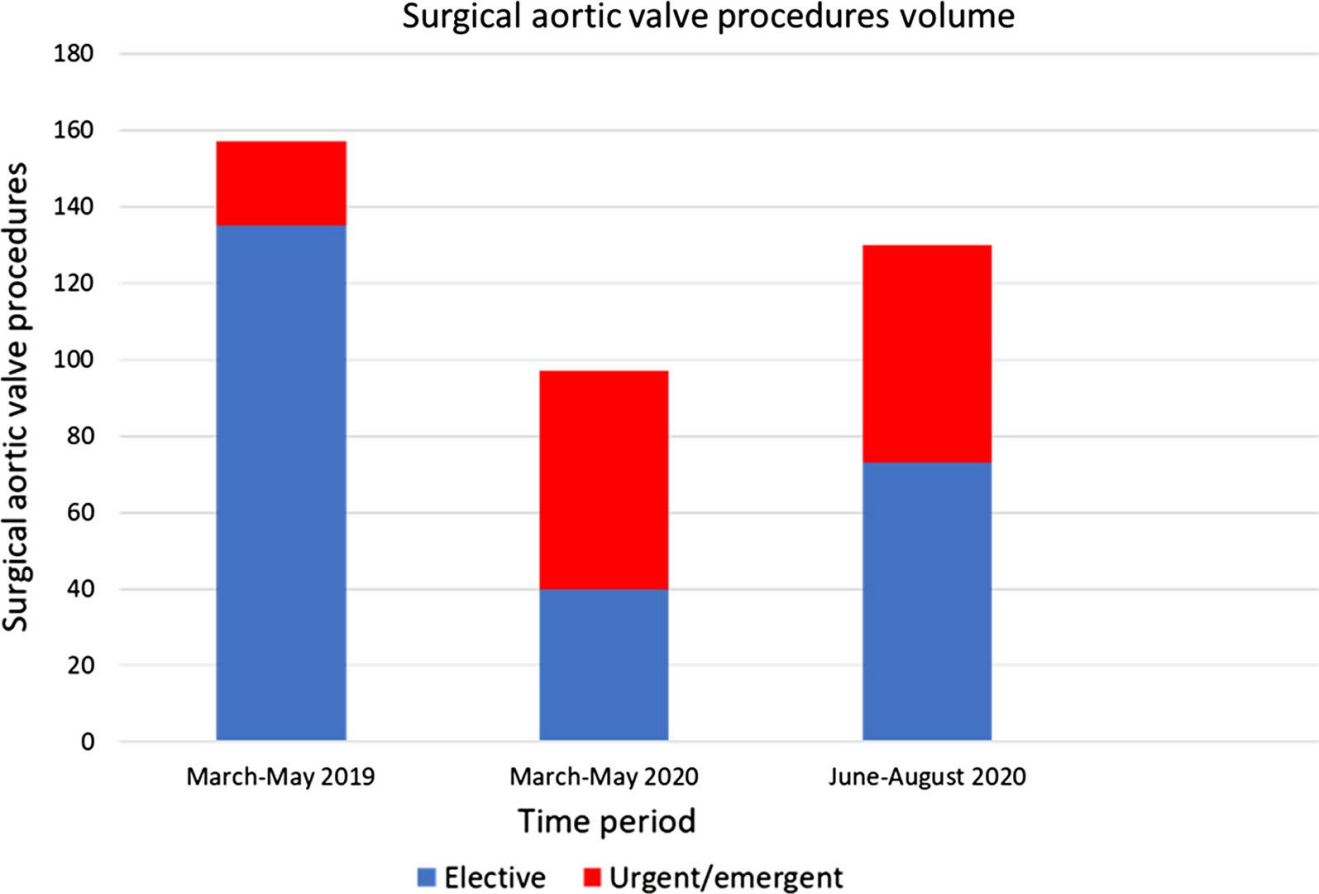
Surgical aortic valve procedures according to time period

During March–May 2020 a total of 97 aortic valve surgical procedures were undertaken, versus 157 during the respective 2019 period (Table 1, Fig. 1). 59% of the patients were operated on an urgent or emergency setting in 2020, versus 14% in 2019 (*p* < 0.001; Table 1, Fig. 1). There was a 70% decrease in elective cases in 2020, in contrast with a 159% increase in urgent/emergency cases (*p* < 0.001; Fig. 1). Similarly, 11.3% of the operations were for aortic valve endocarditis in 2020, versus 4.5% in 2019 (*p* = 0.038). The proportion of re-do operations did not significantly differ (8.2% in 2020 versus 3.8% in 2019, *p* = 0.135) and neither did the percentage of cases classified as New York Heart Association (NYHA) class III/IV and/or Canadian Cardiovascular Society (CCS) class III/IV (76.3% in 2020 compared with 64.3% in 2019, *p* = 0.112). Median Euroscore II was 2.7 [1.9–5.2] in 2020 versus 2.1 [0.9–3.7] in 2019 (*p* = 0.005).

**Table 1:** Number and type of operations, risk assessment, and major post-operative complications during the initial pandemic response compared to the pre-COVID-19 era

Period	March–May 2019 (n = 157)	March–May 2020 (n = 97)	*p*-value
Age, mean (SD)	65.5 (13.4)	66.5 (13.5)	0.45
Male	105 (66.8%)	66 (68%)	0.34
*Ethnicity*			
White	117 (75%)	74 (76%)	0.3
Asian	5 (3%)	2 (2%)	0.2
Black	5 (3%)	2 (2%)	0.46
Other	30 (12%)	19 (20%)	0.23
Ischaemic heart disease	34 (21.7%)	18 (18.6%)	0.274
Chronic lung disease	23 (14.6%)	17 (17.5%)	0.48
Hypertension	96 (61.1%)	62 (63.9%)	0.52
Diabetes	29 (18.5%)	19 (19.6%)	0.28
Dyslipidaemia	57 (36.3%)	30 (30.9%)	0.56
Chronic kidney disease	14 (8.9%)	12 (12.3%)	0.25
Coagulopathy	7 (4.5%)	3 (3.1%)	0.6
Chronic liver disease	3 (1.9%)	2 (2.1%)	0.34
Malignancy	2 (1.3%)	1 (1%)	0.65
*Type of operations*			
AVR	77	52	N/A
AVR + CABG	32	15	N/A
AVR + aortic	29	17	N/A
AVR + MVR	8	3	N/A
Multivalvular/complex	11	10	N/A
Euroscore II (median [IQR])	2.1 [0.9–3.7]	2.7 [1.9–5.2]	0.005
Urgent/emergency setting	22 (14%)	57 (59%)	< 0.001
NYHA/CCS III/IV	101 (64.3%)	74 (76.3%)	0.112
Endocarditis	7 (4.5%)	11 (11.3%)	0.038
Re-do operations	6 (3.8%)	8 (8.2%)	0.135
30-day mortality	1 (0.6%)	0 (0%)	0.58
Re-sternotomy for bleeding	10 (6.4%)	3 (3.1%)	0.269
TIA/stroke	3 (1.9%)	2 (2.1%)	0.54
New need for RRT	8 (5.1%)	9 (9.3%)	0.186
Pre-operative COVID-19	N/A	0 (0%)	N/A
Post-operative COVID-19	N/A	0 (0%)	N/A

During the initial pandemic response there was a 38.2% reduction in the total number of operations with a 70% reduction in elective cases, compared with a 159% increase in urgent and emergency operations. Recommencement of activity at both institutional sites conferred a surgical volume within 17% of the pre-COVID-19 era.

Importantly, despite the higher surgical risk of cases in 2020, the frequency of the investigated major post-operative complications was not adversely affected. More specifically, no fatalities within 30 days were reported, compared with one fatality in the respective 2019 period (*p* = 0.58). Similarly, the incidence of re-sternotomy for bleeding or tamponade was 3.1% versus 6.4% (*p* = 0.269), while the incidence of post-operative neurologic impairment in the form of TIA or stroke was 2.1% versus 1.9% (*p* = 0.54), for the 2020 compared to the 2019 period, respectively. The incidence of renal dysfunction necessitating RRT was 9.3% during the outbreak, versus 5.1% (*p* = 0.186) during the respective 2019 period. All of our patients had a negative pre- and post-operative COVID-19 status.

During June–August 2020, following resumption of surgical activity at both sites, a total of 130 aortic valve procedures were performed, compared with 97 during the first wave period (Table 2, Fig. 1). 43% of the patients were operated on an urgent or emergency setting during June–August, versus 59% during the first wave (*p* = 0.026; Table 2, Fig. 1). In more detail, the absolute number of urgent or emergency cases did not change, in contrast with an 82% increase in the elective cases (*p* < 0.001). In addition, there was a significant increase in the relative frequency of redo-operations to 18.4% (*p* = 0.04; Table 2). No significant difference was detected in either the median Euroscore II risk classification, or any of the investigated post-operative complications (Table 2). Similar to the first pandemic period, the pre- and post-operative COVID-19 status of all of our patients was negative.

**Table 2:** Number and type of operations, risk assessment, and major post-operative complications progression following recommencement of surgery at both trust sites

Period	March–May 2020 (n = 97)	June–August 2020 (n = 130)	*p*-value
Age, mean (SD)	66.5 (13.5)	67.4 (13.7)	0.683
Male	66 (68%)	89 (68.4%)	0.74
*Ethnicity*			
White	74 (76%)	94 (72%)	0.654
Asian	2 (2%)	4 (3%)	0.43
Black	2 (2%)	4 (3%)	0.231
Other	19 (20%)	28 (22%)	0.343
Ischaemic heart disease	17 (17.5%)	20 (15.4%)	0.245
Chronic lung disease	17 (17.5%)	21 (16.2%)	0.62
Hypertension	62 (63.9%)	83 (63.8%)	0.544
Diabetes	19 (19.6%)	23 (17.7%)	0.4
Dyslipidaemia	30 (30.9%)	42 (32.3%)	0.254
Chronic kidney disease	12 (12.3%)	10 (7.6%)	0.37
Coagulopathy	3 (3.1%)	5 (3.8%)	0.5
Chronic liver disease	2 (2.1%)	3 (2.3%)	0.661
Malignancy	1 (1%)	2 (1.5%)	0.225
*Type of operations*			
AVR	52	61	N/A
AVR + CABG	15	18	N/A
AVR + aortic	17	29	N/A
AVR + MVR	3	8	N/A
Multivalvular/complex	10	14	N/A
Euroscore II (median [IQR])	2.7 [1.9–5.2]	2.8 [1.5–5.2]	0.469
Urgent/emergency setting	57 (59%)	57 (43%)	0.026
NYHA/CCS III/IV	74 (76.3%)	101 (77.7%)	0.417
Endocarditis	11 (11.3%)	16 (12.3%)	0.55
Re-do operations	8 (8.2%)	24 (18.4%)	0.04
30-day mortality	0 (0%)	3 (2.3%)	0.2
Re-sternotomy for bleeding	3 (3.1%)	10 (7.7%)	0.167
TIA/stroke	2 (2.1%)	4 (3%)	0.38
New need for RRT	9 (9.3%)	7 (5.4%)	0.178
Pre-operative COVID-19	0 (0%)	0 (0%)	0.628
Post-operative COVID-19	0 (0%)	0 (0%)	0.628

## Discussion

In this service evaluation project, we have demonstrated that the COVID-19 pandemic resulted in a significant decrease in the total number of conducted aortic valvular operations in our trust. This decrease was mitigated by the expansion of surgical activity over both of our trust hospital sites. The prioritization of severe aortic valve disease cases leaded to a relative increase of urgent and emergent operations with an attendant enhanced operative risk. Importantly, neither mortality nor major post-operative complications rate increased. Furthermore, our stringent pre-operative COVID-19 screening protocol prevented contraction of COVID-19 among our surgical cohort.

The intensity of COVID-19 had a major impact on the provision of surgical services worldwide [3–6]. The perioperative dependence of cardiac surgery patients on Intensive Care Unit (ICU) care, on which a significant component of the pandemic response was placed, and the concomitant reallocation of staff and equipment particularly complicated their management [6, 12, 13]. In this context, Pan-London Emergency Cardiac Surgery (PLECS) service was formed to provide a centralised pathway for urgent and emergency cases in London [7]. Royal Brompton and Harefield trust was one of the two centres selected, based on its surgical capacity, location, and absence of Accident & Emergency department. These characteristics provided the capability of accommodating high surgical volumes in a COVID-19 free environment.

Our modus operandi resembled the guidelines pertaining to cardiac surgery services during the pandemic response, issued by the Lombardi Region [14]. In specific, a hub-and-spoke system was implemented: Harefield Hospital site played the hub role during the first pandemic period (March–May 2020) allowing continuation of operations via a common referral pathway, while Royal Brompton served as a spoke component, temporarily withholding surgical activity in order to accommodate the increasing ECMO referrals. The expansion of critical care bed availability and the COVID-19 status-based compartmentalisation allowed the continuation of operations at both trust sites during the second pandemic period (June–August 2020), while minimising the COVID-19 contraction risk.

To this end, a stringent admission protocol was utilised. All patients were screened with 2 serial COVID-19 swabs, one taken within 72 h of admission and a second taken on admission (within 48 h of their predicted operative date). Patients at home would need to shield completely for 14 days and would have a pre-admission workup including a COVID-19 swab obtained 3 days prior to admission. They were subsequently admitted 2 days prior to their surgery with a COVID-19 risk determined as ‘GREEN’ (COVID-19-negative). Patients transferred from other hospitals would only be transferred to our institution with a negative COVID-19 swab obtained within 72 h of transfer. As these patients had not been shielding, they were treated as potentially COVID-19 positive (‘AMBER’) and were barrier nursed in-side rooms until their status could be determined. All patients had a CT scan performed in the immediate pre-operative period (1–2 days before the provisional operation date). A positive swab or any suspicious radiological findings would place the patient in the ‘RED’ risk group (COVID-19-positive) and would be an indication to defer the operation; in the interim the patient would be under the care of Respiratory Medicine until 2 negative COVID-19 swabs were provided. As a result, none of our patients contracted the disease over the investigated period, underlining the effectiveness of this protocol. This has important clinical implications as signified by a recent study including nine United Kingdom (UK)-based cardiac surgery centres, where COVID-19 diagnosis was independently associated with a 21% increase of in-hospital mortality and a prolongation of median length of stay by 6 days [15].

The COVID-19 pandemic resulted in a significant reduction of cardiac surgery operations worldwide. According to a recent survey that included 60 cardiac surgical centres globally, there was a median reduction of 50–75%, while most of the contributing hospitals abandoned the provision of elective care. In 5% of the centres, all surgical activity including emergency operations was withheld. Importantly, these detrimental effects were similarly evident in high- and low-volume centres [12]. Similarly, national-wide in the UK an 83% reduction in index cardiac cases over the March–May 2020 period was documented [6]. An interesting study that extracted national-wide data from the Hospital Episode Statistics National Health Service (NHS) database demonstrated that surgical AVR was among the most intensely affected procedures, with a 91% decrease in cases performed during March–May 2020 compared with the mean number of operations conducted over the respective 2018 and 2019 period [16]. In the United States (US), the pandemic caused a reduction in cardiac surgery cases by 53%: elective cases were reduced by 65%, but non-elective operations were also significantly affected with a decrease of 40%. These effects were enhanced in Mid-Atlantic region, where a decline of 71% was documented [17]. Even in countries where COVID-19 was initially contained due to strict restrictive measures, valvular heart disease operations were among the most severely affected procedures: 75% reduction in surgical caseload was reported by two large Greece-based centres [18]. On the other hand, an aortovascular disease centre in the UK managed to maintain its surgical volume during the investigated outbreak period [13]. Moreover, a self-explanatory increase in the proportion of emergency and urgent cases has been demonstrated. While most of the studied centres abandoned the provision of elective surgical care [12], a large UK-based aortovascular centre equally distributed its operations between elective and emergency care [13]. Other hospitals experienced doubling of their emergency cases relative caseload to 32.1% [18]. Our trust appears to have performed non-inferiorly, demonstrating a 38% initial reduction of AVR caseload, which was mitigated by the resumption of activity across both trust sites to 17%. Urgent and emergent cases constituted the greatest component of our workload over the first pandemic period, with an even distribution over the June–August 2020 period.

A national-wide UK registry report revealed that mortality of surgical AVR performed during the first pandemic period was not compromised, despite the relative increase of urgent/emergent cases and the attendant increased risk [16]. However, COVID-19 per se exerted detrimental effects by way of a 21% increase in mortality and a significant prolongation of length of stay [15]. A US-based study that obtained data from the Society of Thoracic Surgery (STS) Adult Cardiac Surgery Database demonstrated a detrimental effect of the pandemic on surgical mortality. In specific, the observed-to-expected (O/E) mortality ratio in the Mid-Atlantic and New England regions rose to 1.2 for isolated coronary artery bypass graft (CABG) cases, corresponding to an increase of 167%; the respective increase for all cardiac surgery procedures was 110% [17]. These observations were attributed to patients presenting at more advanced disease states leading to an augmented non-elective operations relative frequency with an attendant increase in surgical risk. Our unit managed to preserve the pre-COVID-19 surge mortality and major post-operative complications rate despite the significantly increased risk; the efficiency of our COVID-19 screening protocol appears to have contributed significantly in the light of the above-mentioned study findings [15].

Despite the apparent non-inferiority in the quantity and quality of our cardiac surgical services during the pandemic response, the 70% initial reduction in elective activity suggests the development of a significant backlog of cases; this was mitigated by the resumption of operative activity at both institutional sites, which conferred a significant increase of elective activity by 82%. The backlog may mainly include patients with asymptomatic or mildly symptomatic disease; however, given the non-negligible occurrence of sudden cardiac death even in asymptomatic patients with advanced aortic valvular disease (especially aortic stenosis [11]), following normalisation of operative activity across both sites, these cases should optimally be accommodated to minimise the possibility of any preventable deaths. This is further highlighted by evidence form healthcare systems of routine limited capacity, where longer cardiac surgical waitlists have been associated with worse operative mortality [19].

## Conclusions

Our aortic valve surgical services were significantly affected by the COVID-19 pandemic, resulting in prioritization of urgent and emergency cases and deferral of elective treatment. Despite the increased attendant surgical risk, perioperative mortality and major morbidity were not increased. It would be of interest to follow-up patients treated during the pandemic and investigate for longer-term consequences as well as to evaluate how the backlog of elective cases will be accommodated after normalisation of surgical activity.

## Data Availability

The data that support the findings of this study are available on reasonable request from the corresponding author. The data are not publicly available due to privacy or ethical restrictions.

## Abbreviations

AVR: Aortic valve replacement
CABG: Coronary artery bypass surgery
CCS: Canadian Cardiovascular Society angina pectoris grading
COVID-19: Coronavirus-disease 2019
ECMO: Extracorporeal Membrane Oxygenation
ICU: Intensive care unit
MVR: Mitral valve replacement/repair
NHS: National Health Service
NYHA: New York Heart Association functional classification
PLECS: Pan-London Emergency Cardiac Surgery
RRT: Renal replacement therapy
TIA: Transient ischaemic attack
UK: United Kingdom
US: United States
